# Negative phototaxis of jumping cocooned parasitoid wasp larvae against short wavelengths and physicochemical properties of the cocoon shell

**DOI:** 10.1038/s41598-023-36686-0

**Published:** 2023-06-12

**Authors:** Shun-ichiro Iwase, Midori Tuda, Yuma Sugawara, Katsuto Fukuda, James R. Miksanek, Midori Watanabe

**Affiliations:** 1 Institute of Biological Control, Faculty of Agriculture, Kyushu University, Fukuoka, 819-0395 Japan; 2 Laboratory of Insect Natural Enemies, Department of Bioresource Sciences, Faculty of Agriculture, Kyushu University, Fukuoka, 819-0395 Japan; 3 Center of Advanced Instrumental Analysis, Kyushu University, Fukuoka, Japan; 4 Present Address: Research Institute of Environment, Agriculture and Fisheries, Habikino, Osaka Prefecture Japan

**Keywords:** Agroecology, Animal migration, Behavioural ecology

## Abstract

The parasitoid wasp *Bathyplectes anurus* (Hymenoptera: Ichneumonidae: Campopleginae) is a successful biocontrol agent against the alfalfa weevil, *Hypera postica*. This weevil is a serious pest of beneficial fabaceous plants such as alfalfa and Chinese milk vetch. One of the possible reasons for the success of this wasp in hot climates may be the ability of its cocooned larvae to repeatedly jump and roll until they relocate themselves away from detrimental sunlight and heat. It is not yet known which wavelengths of light trigger this avoidance behavior or the microstructure of the cocoon shell that might allow light transmission. Here, the response of the cocooned larvae to different wavelengths, and the microstructure, hardness, and elemental components of the cocoon shell were studied. A population of cocooned larvae were introduced on the boundary line between illuminated and shaded areas with blue, green, red, or near-infrared light-emitting diodes. The cocoons moved away from the blue and green light. The distance from the boundary to the cocoons in the shaded area was longer under these short wavelengths, followed by the red light and shortest under the near-infrared light and nil under darkness. No difference was found in mortality between different wavelengths after three days of illumination. Scanning electron microscope observations of the surface of the cocoon shell revealed that the belt-like central ridge was porous, which likely allows ventilation and light transmission. The surface of the cocoon shell showed a uniform distribution of sulfur, potentially aiding in the capture of green wavelengths. The ridge had twice the thickness of the main body and was 1.9 times harder than the main body. These results may be applied to better understand the individual responses of this biological control agent to modifications to their environment, including light pollution.

## Introduction

The responses of adult insects towards different wavelengths of light has been well studied.s Many insects exhibit positive phototaxis and are attracted to short wavelengths of light such as green, blue, violet, and ultraviolet (UV)^1–11^; others may respond to longer wavelengths like red and yellow light^12,13^. By contrast, the limited mobility of the immature stages of terrestrial invertebrates have drawn less attention in studies of taxis towards or away from specific wavelengths of light (except for aphids^14^, midges^15^, fruit flies^16^, beetles^17,18^ and moths^19^), even though their vulnerability or behavioral response to light may differ from that of adults^20^.

Despite the relatively sessile nature of the immature stages of many insects, some are highly mobile. Jumping, a rare locomotor mode of immature insects, is observed in flies and wasps^21–24^, such as some parasitic wasp species in a few genera of the subfamily Campopleginae (Hymenoptera: Ichneumonidae). Wasps in these genera are solitary koinobiont endoparasitoids; a single parasitoid larva develops inside its host while host development continues (before ultimately leading to the death of the host). The cocooned larvae of some campoplegine species jump to avoid unfavorable abiotic environments or attacks by predators and other parasitoids^21,24^. Jumping followed by rolling may be rapid and energy-saving dispersal behavior relative to creeping locomotion^21–24^.

**Figure 1:**
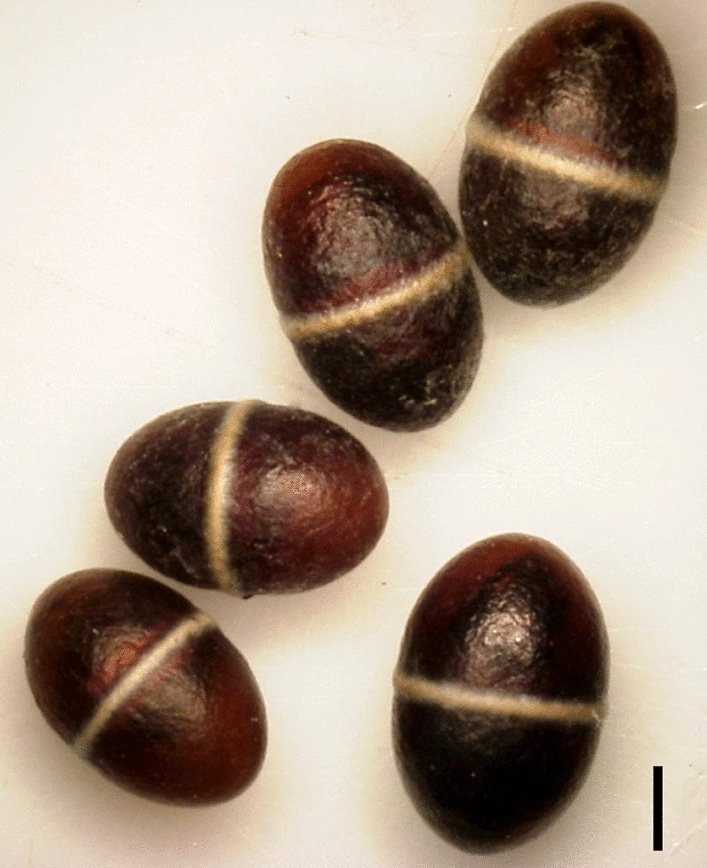
Cocooned *Bathyplectes anurus* larvae, with white central ridge. The scale, 1 mm.

*Bathyplectes anurus* (Thomson) (Hymenoptera: Ichneumonidae: Campopleginae) is used as one of the biological control agents of the alfalfa weevil [*Hypera postica* (Gyllenhal) (Coleoptera: Curculionidae)], a serious pest of beneficial fabaceous plants such as alfalfa and Chinese milk vetch^25,26^. Adult females lay eggs in weevil larvae. The parasitoid larva feeds on, eventually kills, and crawls out from the host larva and then forms a cocoon inside the host cocoon spun at its last instar larva. These parasitoid cocoons are oval-shaped and dark brown with a slightly raised, white, belt-like ridge in the middle (Fig. 1). When the parasitoid larva in the cocoon twitches, the entire cocoon jumps approximately 5 cm, followed by landing and rolling. The jumping and rolling itself may be in a random direction, but these behaviors are repeated until the cocooned larva reaches a microhabitat with favorable conditions. Cocooned larvae avoid light, among other unfavorable environmental conditions or predatory risks, before being rendered immobile after pupation in the summer^21,24^.

The specific light wavelengths that *B. anurus* larvae respond to and avoid are not yet known, which might affect its biological control efficiency in the vicinity of urban settings where nocturnal light pollution is anticipated^27,28^. The successful establishment and spread of *B. anurus* in the field has been variable and may partly depend on local field conditions disturbed by urbanization^29,30^. Here, we aim to test whether cocooned larvae of the parasitoid *B. anurus* disperse away from specific ranges of wavelengths of light. We tested this phototaxic behavior using a bioassay that incorporated four different types of light-emitting diodes (LEDs). Finally, to investigate the potential mechanism for light transmission through the cocoon, the surface microstructure, elemental composition, and hardness of the cocoons were studied using a scanning electron microscope (SEM), an energy-dispersive X-ray (EDX) spectrometer, and a rheometer.

## Materials and methods

### Material

For the experiment on the immediate response of parasitoid cocoons to light, we used the cocooned larvae of *Bathyplectes anurus* (Fig. 1) emerged from alfalfa weevils, *H. postica*, collected from the fabaceous herbs *Astragalus sinicus* and *Vicia sativa* subsp. *nigra* (syn. *V. angustifolia*) in May 2017 in Fukuoka Prefecture, Japan. These insects were kept at room temperature (approximately 25 °C) and under complete darkness. The first experiment was performed in mid-June 2017. For the survival experiment, we used cocooned larvae collected from the same host plants in late April through early May in 2018 in Fukuoka Prefecture. They were stored under the same conditions as those from 2017. This experiment was performed from early May to mid-June 2018. Prior to both experiments, the jumping abilities of all cocooned larvae were confirmed, and those that did not jump were not used in the experiments. The cocoons were then stored in a plastic cup covered with aluminum foil in a dark incubator at 25 °C and 55–60% r.h. for 48 h before each experiment.

### Response to light color

**Figure 2:**
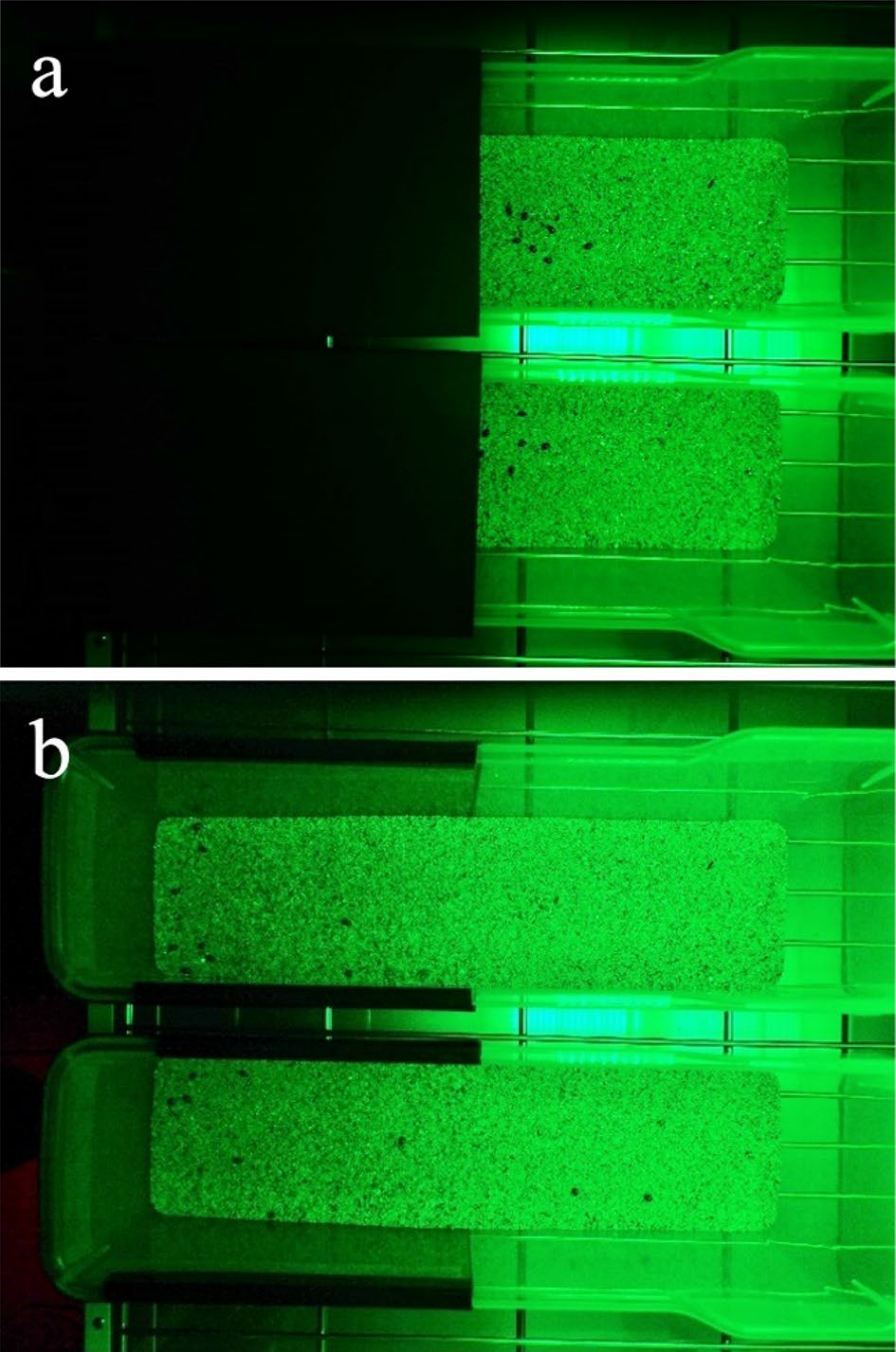
A representative top view of the two experimental arenas for green LED illumination, (**a**) within 5 s of the beginning of illumination (with half area covered), and (**b**) after 20 min (when the cover was removed).

Half (13 cm × 7 cm, 7 cm height) of a clear plastic container (26 cm × 7 cm, 7 cm height) was wrapped in black paper to exclude light (Fig. 2). The bottom of the container was filled with beige-colored sand (Bottom Sand S-8810, Sudo, Nagoya, Japan) approximately 8 mm in depth. LEDs were illuminated 27 cm above the sand surface in a dark room with a controlled ambient temperature of 25 °C and 52–59% r.h. Ten randomly selected cocoons were placed on the center line that divided the illuminated half, in which the intensity of light was uniform, from the shaded half, in which the light intensity diminished with distance from the center (Fig. 2). Ten replicated populations were subjected to each of the following LED illumination treatments: blue (peak wavelength of 470 nm), green (525 nm), red (660 nm) and near-infrared (735 nm) (ISL-150 × 150, CCS Inc., Kyoto, Japan) at 10.5 μmol m^−2^ s^−1^ of photosynthetic photon flux density (PPFD) (corresponding to 777 lx). Each population was used only once and a total of 420 cocooned larvae were used in the experiment. For six out of the 10 replicated populations for each LED, the cocoons on the illuminated side were photographed from above at 5, 10, 15 and 20 min after the cocoon introduction, without additional light. At 20 min, both sides were photographed with the black paper cover removed. For the rest of the replicated populations, the cocoons on the illuminated side were photographed likewise every 60 s for the first 10 min. As a control, three replicated populations of six or seven cocoons were subjected to total darkness and photographed during a brief exposure (< 2 s) to a dim light at 10 and 20 min. The cocooned larvae on the illuminated side were counted. The cocoons lying on the center line were counted as 0.5. Finally, to quantify preference for light intensity, the distance from the center line to each cocoon on the shaded side at 20 min was measured.

### Effect of light color on wasp survival

To evaluate the effect of the different wavelengths of light on the survivorship of cocooned larvae, we illuminated each of three populations consisting of seven cocoons in plastic cups (10 cm diameter) with one of the four different LEDs for 72 h or under darkness (control). After exposure, the cocoons were placed under darkness for 35 d before being illuminated with a white LED at 15 PPFD for a maximum of 20 min to check for jumping ability. Immobile cocoons were then dissected to verify the death of the larvae.

### Microstructure, elemental composition, and hardness of cocoon shell

The microstructure of the surface of the cocoon shell (control cocoons kept under darkness) was investigated using SEM (TM4000Plus, Hitachi High-Tech, Tokyo, Japan) at an accelerating voltage 10 kV or 15 kV and at × 80, × 180, or × 500 magnification. Finer structures of shell surface as well as cross-sections were also examined using SEM (JSM-IT700HR, JEOL Ltd., Tokyo, Japan) at a 10 kV accelerating voltage and at × 400 or × 500 magnification. Additionally, to visualize the structure of different regions of the cocoons in cross-section nondestructively, two cocoons were scanned using 3D micro-CT (SKYSCAN 1272 with CMOS detector, Bruker).

The elemental composition of the surfaces of the three control cocoons (kept under darkness) was inspected using an EDX spectrometer (Quantax75, Bruker, Billerica, MA, USA) attached to SEM (TM4000Plus) at an accelerating voltage of 15 kV and at × 180 magnification, and the most representative cocoon was chosen for a detailed analysis.

FTIR spectra of the samples were done on a Fourier transform infrared spectrometer (FT/IR-6800, JASCO, Tokyo, Japan). To compare chemical profile between different regions of the outer surface of cocoons, we applied an attenuated total reflection (ATR) method. Obtained profiles were compared with unprocessed silk and calcium oxalate (CaC_2_O_4_).

The hardness (measured in Newtons, N) of four cocoons per light color treatment was measured using a rheometer (Compac-100, Sun Scientific Co., Tokyo, Japan) at a stress speed of 60 mm min^-1^.

### Statistics

A general linear model was applied to the proportion of cocoons on the illuminated side, with light color/darkness treatment, time from introduction, the interaction between light color/darkness and time, and population identity nested within light color/darkness treatment as explanatory variables. A general linear model was also applied to the distance of the cocoons from the center line, with light color/darkness treatment and population identity nested within light color/darkness treatment as explanatory variables. For the survival experiments, the effect of light color on survival rate was tested with a logistic model. The thickness of the shell of the cocoon in different areas (brown body vs. white central ridge) as well as the width of the fibers in different orientations within the white ridge were compared with nonparametric Wilcoxon tests. The hardness of the cocoons (ln-transformed) was tested with a general linear model with light color/darkness treatment, area (brown body or white central ridge), their interaction, and replicate nested within light color/darkness treatment. For general linear models, we confirmed the normality of residual distributions, and a posthoc Tukey-type test was performed for multiple groups with statistical significance at *p* = 0.05.

## Results

### Response to light color

**Figure 3:**
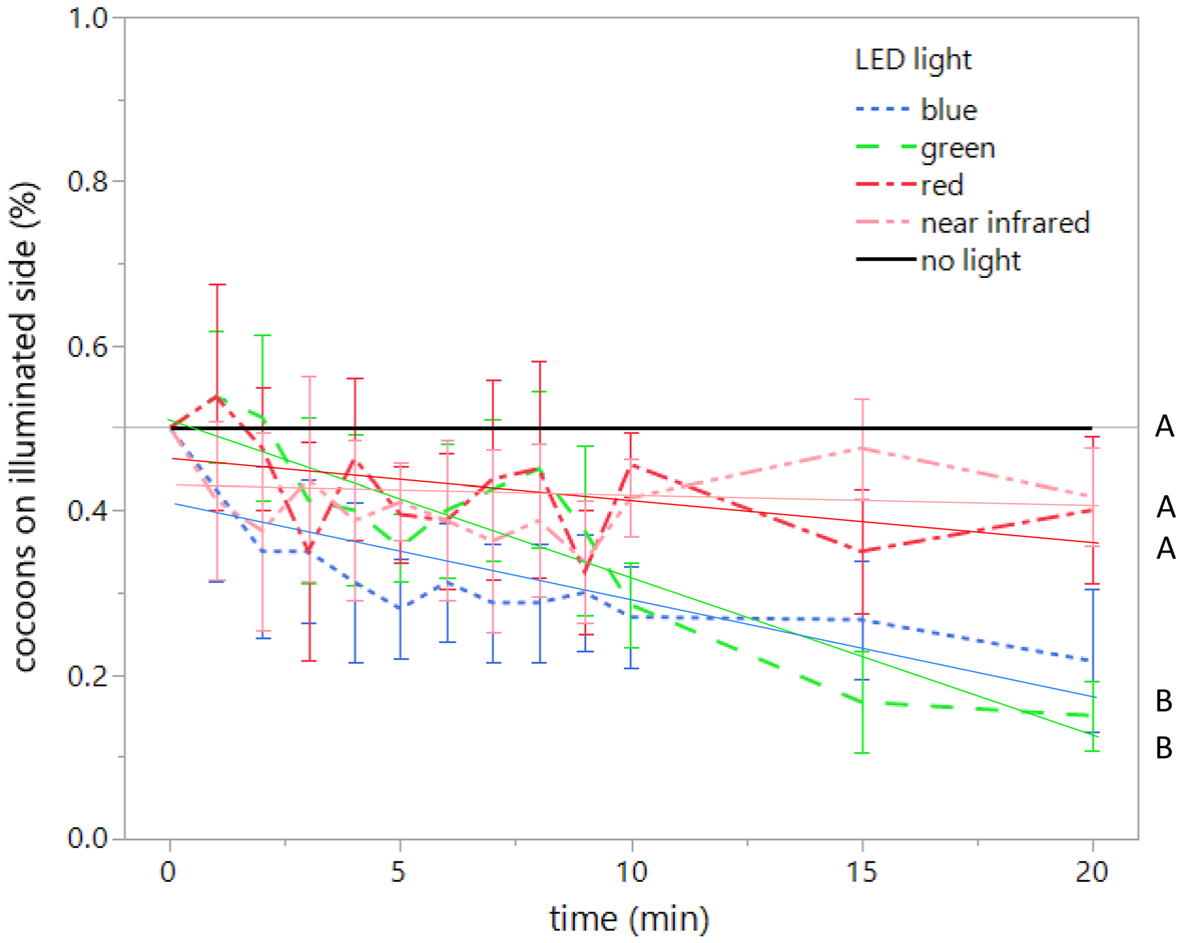
The proportions of cocooned *Bathyplectes anurus* larvae on the illuminated side over time under the lights with different wavelengths or no light. Shared capital letters show nonsignificant differences among wavelengths or no light treatment. Linear regression lines with 95% confidence intervals.

The proportion of cocoons on the illuminated side was affected by light color (Table 1, Fig. 3); fewer cocoons were found under the blue or green light than under the red and near-infrared light (Fig. 2). There was a general trend of the cocoons jumping away from the light over time (Table 1, Fig. 3) and an interaction effect was found between light color and time (Table 1). There was a significant variation among populations, nested within light color (Table 1).

**Table 1:** Test results of effects of light color and time on the proportion of cocooned larvae on the illuminated side.

	df	*F*	*p*
Light color	4, 260	17.75	< 0.001
Time	1, 260	46.31	< 0.001
Time × light color	4, 260	5.91	< 0.001
Population [light color]	38, 260	12.59	< 0.001

Population identity was nested within light color.

**Figure 4:**
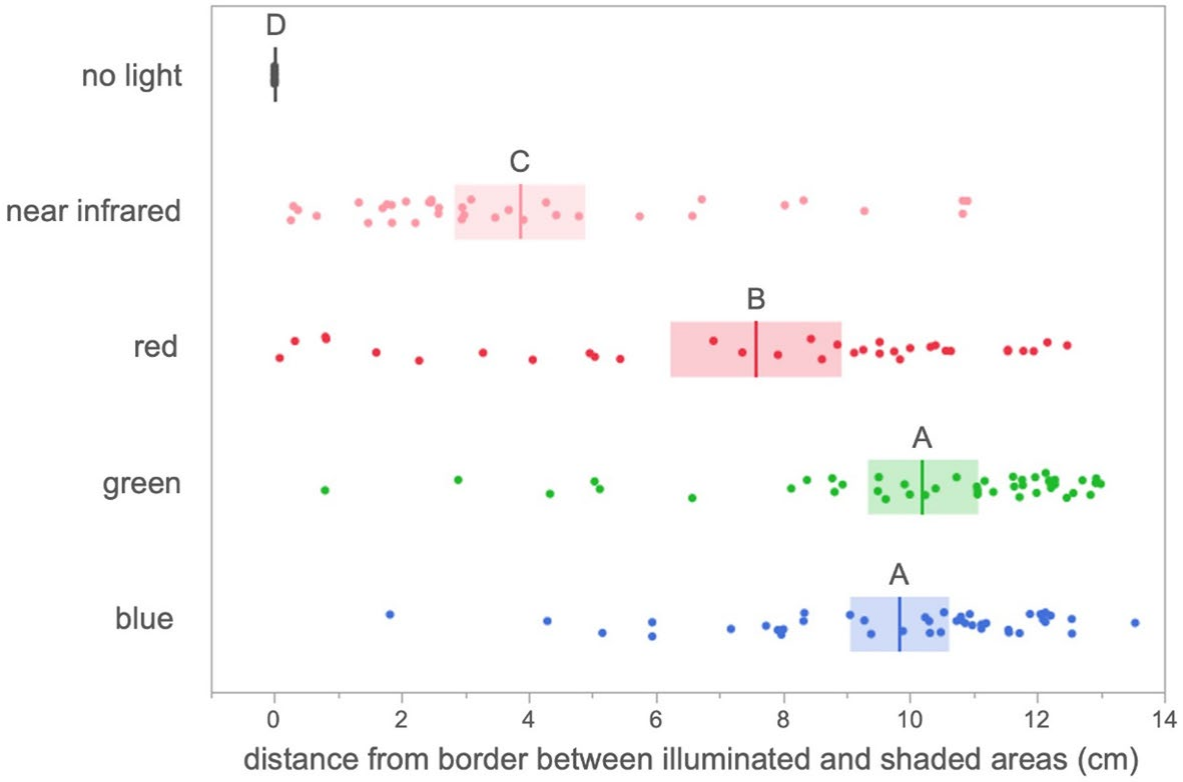
Distance (least square mean + SE) of cocooned *Bathyplectes anurus* larvae in shaded area from the border (where the cocoons were released) between illuminated and shaded areas at 20 min under the light with different wavelengths or no light. Shared capital letters show nonsignificant differences among wavelengths or no light treatment. Means with 95% confidence intervals.

For the cocoons that dispersed to the shaded side after 20 min had elapsed, the net travel distance from the center boundary between the illuminated and shaded areas was significantly different among different light colors or darkness (*F* = 62.99, d.f. = 4, *p* < 0.0001), with no difference among populations (nested within light color/darkness, *F* = 0.82, d.f. = 22, *p* = 0.695). The distance was shortest under the near-infrared light (3.85 ± 0.51 cm, mean ± SE, *n* = 37), followed by that under the red light (7.56 ± 0.66 cm, *n* = 34), and was longest under the blue (9.82 ± 0.39 cm, *n* = 42) and green lights (10.19 ± 0.43 cm, *n* = 44) (Figs. 3 and 4). Under complete darkness, none of the cocoons showed horizontal dispersal (Fig. 4).

### Effect of light color on wasp survival

There was no difference in survival rate among larvae previously illuminated by different colors or put under darkness (likelihood ratio* χ*^2^ = 3.26, d.f. = 4, *p* = 0.516). The only larva found dead was under the blue illumination treatment (survival probability of 0.952 for 38 d).

### Microstructure, elemental composition, and hardness of cocoon shell

**Figure 5:**
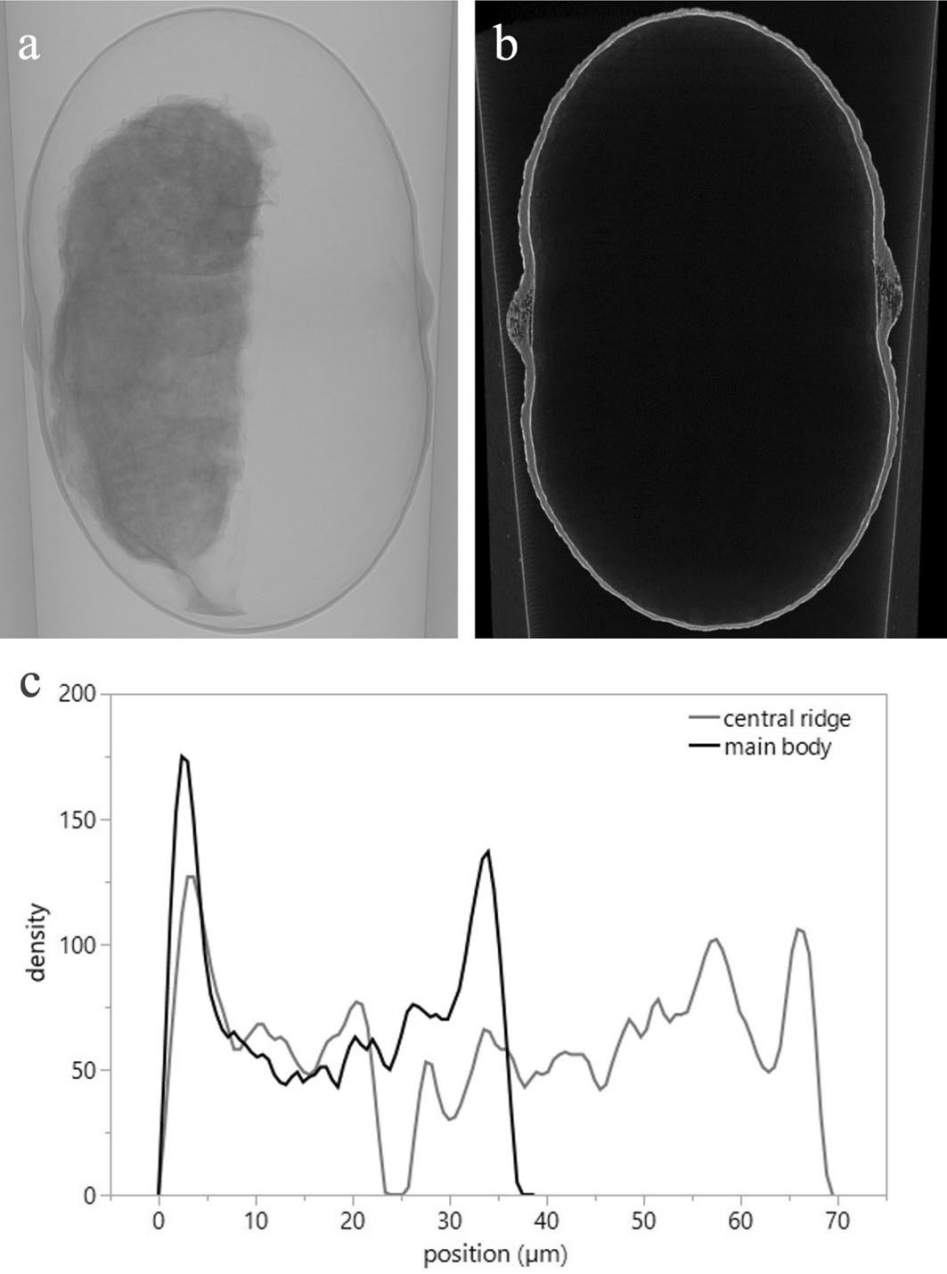
(**a**) X-ray micro-CT projection, (**b**) cross-section images, (**c**) cross-section density distribution in main body and central ridge of a cocoon shell of *Bathyplectes anurus*.

**Figure 6:**
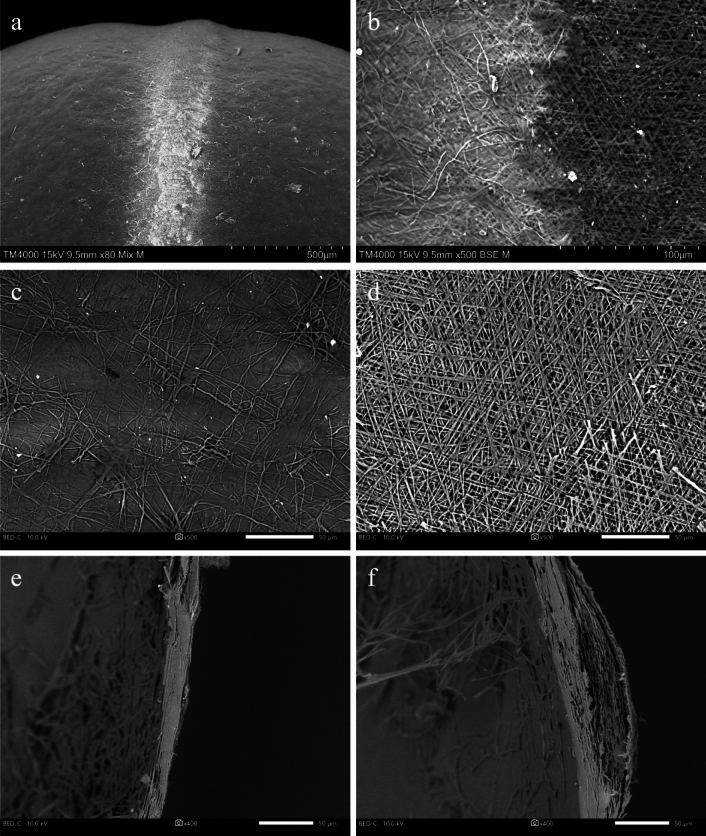
Scanning electron microscopy (SEM) images of surface microstructure of a cocoon of *Bathyplectes anurus*. (**a**) Lateral view of the area around the central white ridge, (**b**) area between the main body and central ridge, (**c**) main body, (**d**) central ridge, (**e**) cross section of main body, (**f**) cross section of central ridge.

**Figure 7:**
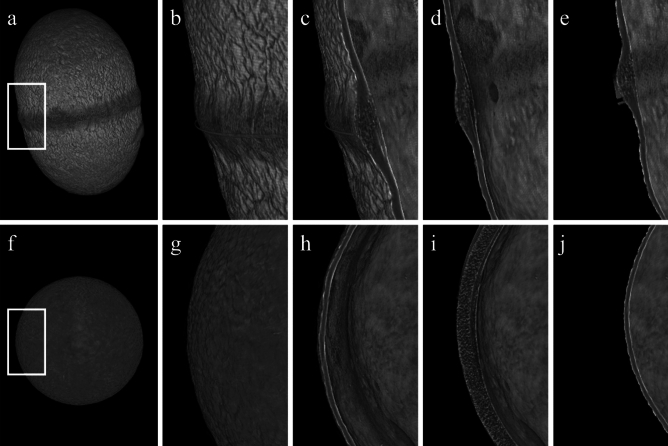
Micro-CT imaging of a representative jumping wasp cocoon, showing (**a**–**e**) longitudinal sections and (**f**–**j**) transverse sections. (**a**) Exterior surface view of the whole cocoon, with the region of interest indicated by the white box. (**b**) Exterior surface view. (**c**) Upper-mid cross-section. (**d**) Cross-section through a central plane. (**e**) Lower-mid cross-section. (**f**) Exterior surface view of the whole cocoon, with the region of interest indicated by the white box. (**g**) Exterior surface view. (**h**) Upper-mid cross-section. (**i**) Cross-section through a central ridge. (**j**) Lower-mid cross-section. All images are to scale.

The central ridge was 2.0 times thicker than the main body of the cocoon wall (Fig. 5a,b, *χ*^2^ = 10.5, d.f. = 1, *p* = 0.001) [central ridge: 76.9 ± 5.0 µm (mean ± SD); main body: 38.6 ± 5.2 µm]. A reading of the SEM and micro-CT images and a density analysis of a cross section of the cocoon showed sparsity of the shell in the central ridge compared to the main body (Figs. 5c, 6e,f and 7c–e, h–j). The central ridge and transitional area toward the main body of the cocoons was composed of fibers spun in three major directions and the surface was uncoated (Fig. 6b,d). The main body of the cocoons was also composed of fibers but was uniformly coated (probably with the glue protein, sericin^31^) (Figs. 6a,c and 7a,b,f,g). The width of the fibers in the central ridge did not differ based on orientations (*χ*^2^ = 1.51, d.f. = 1, *p* = 0.220) and were 1.29 ± 0.525 µm (mean ± SD) (median 1.16, range 0.65–3.17 µm, *n* = 60).

Both the central white ridge and the main body of the cocoon had similar elemental composition; carbon, C (atom density ± absolute error (1σ %) of main body: 59.6 ± 6.7; ridge: 63.9 ± 7.3), oxygen, O (main body: 31.5 ± 5.5; ridge: 27.5 ± 5.1), nitrogen, N (main body: 8.8 ± 2.3; ridge: 8.4 ± 2.4), sulfur, S (main body: 0.06 ± 0.04; ridge: 0.12 ± 0.04), calcium, Ca (main body: 0.01 ± 0.01; ridge: 0.10 ± 0.05), and potassium, K (main body: 0.03 ± 0.04; ridge: 0.02 ± 0.03). The distributions of these elements were homogenous around the surface of the cocoon (Supplementary Fig. S1).

The FTIR spectra of the cocoon showed similar patterns for both the central ridge and the main body (Supplementary Fig. S2). They were similar to the unprocessed cocoon silk (of *Bombyx mori*), indicating that the *B. anurus* cocoon shell is mainly composed of silk protein. By contrast, the characteristic peaks of calcium oxalate were absent or extremely weak in both regions of the cocoon (Supplementary Fig. S2). The central white ridge was 1.9 times harder than the brown body of the cocoons (central white ridge: 0.263 ± 0.014 N (mean ± SE); brown body: 0.139 ± 0.007 N; *F*_1,136_ = 78.98, *p* < 0.001). There was no difference in hardness among cocoons illuminated by light with different wavelengths for three days (*F*_4,136_ = 1.53, *p* = 0.198). There was no significant interaction between light color and cocoon region (*F*_4,136_ = 0.56, *p* = 0.696). There was no significant difference between replications (*F*_5,136_ = 1.17, *p* = 0.327).

## Discussion

More cocooned *B. anurus* larvae jumped away from the light with short wavelengths—blue and green—than away from the light with longer wavelengths—red and near-infrared (Fig. 3). The cocoons travelled further into the shaded side under the light of short wavelengths than under the longer wavelengths (Fig. 4). In Hymenoptera, visual photoreceptors peak in the UV, blue, and green ranges (λ max = 344 nm, 436 nm, and 544 nm, respectively)^32,33^, and adult wasps respond to these wavelengths^9,10^. Photoreceptors for red have been found in a few large-bodied hymenopteran taxa but not in Ichneumonidae^34^. Because *Bathyplectes anurus* cocooned larvae did avoid long wavelengths (red and near-infrared light), but probably hardly perceive red light and longer wavelengths as light, they may instead perceive the long wavelength, near-infrared radiation, as heat. The larvae could perceive these wavelengths by sensing rising body temperature or by directly sensing heat with sensilla on their body surfaces (as has been observed in ticks^35^). Cocooned *B. anurus* larvae have been demonstrated to avoid fluorescent white light (at 130 lx), increased temperatures (0.0–8.2 °C increase rate per 5 min from the control temperature of 24 °C)^24^ and a high constant temperature (43 °C)^21^. The immediate avoidance of these environmental conditions is adaptive for longer periods because the mortality of the cocooned larvae is much higher under the sun (82.1% at maximum soil temperature ≥ 40.2 °C, full sunlight is 108,000 lx) than under the shade (4.5% at maximum soil temperature ≥ 29.9 °C) during the summer months of August and September^24^. Blue light is known to be lethal for insects^36,37^ and this may be caused by the blue light enhancing the production of reactive oxygen species, such as in microbes and human cells^38,39^. Therefore, the avoidance of blue light directly reduces the lethal effect. However, the lethal effect of green light is much lower than that of blue light (on pathogens)^40^. Green light even induces positive effects on the fertility of beetles and the development of moths and fish^41–43^. The behavioral avoidance of green light may be selected for under the pressure of predatory or parasitic insects that are attracted to green light^1,9,10^. By avoiding light with short wavelengths and the heat produced by near-infrared light, even at the low intensity applied in this study, *B. anurus* larvae may be preparing for the immobile pupal stage to be spent under environmental conditions more suitable for survival.

Decreasing dispersal distance correlated with increasing wavelengths in the long wavelength range (Fig. 3). We cannot exclude a possible contribution of green photoreceptor in partially sensing these long wavelengths as was suggested for leafhoppers^44^. Near-infrared light that may be sensed as heat could attract the cocooned larvae if the temperature under complete shade in this study was below the optimal temperature for the *B. anurus* larvae. Then, the cocooned larvae may have either jumped back towards warmer temperatures produced by the near-infrared light or stopped jumping when they sensed decreasing temperatures.

There appeared to be no negative effects of short wavelengths on the survivorship of wasp larvae in the short term. However, in the long term, while blue light can have a positive effect on the sex ratio (proportion female) of parasitoid offspring (whereas red light can have a negative effect)^45^, the negative effects are potentially greater, because repeated jumping is costly for *B. anurus* larvae^24^. Therefore, we suggest that the elimination or reduction of short wavelengths from light sources near fields could be beneficial for this insect and improve the efficacy of biocontrol against the alfalfa weevil, a serious pest of legumes.

Our EDX analysis on the elemental composition of the surface of *B. anurus* cocoons showed that it was rich in carbon, oxygen, and nitrogen. The cocoon silk protein produced by Campopleginae is known to be rich in serine, alanine and glycine^46^, and the high nitrogen content is likely attributed to large amounts of the fibrous protein, fibroin, which could also explain the observed sulfur content due to the presence of disulfide bonds^47,48^. Since sulfur provides the density of states or emissive trap states for photoexcited electrons, and hence improves absorbance and photoluminescence intensity in the wavelength of 560–570 nm^49^, the sulfur on the cocoon surface might help absorb green light. Additionally, the melanin, eumelanin (made of 5,6-dihydroxyindole C_8_H_7_NO_2_), that appears brown to black is probably responsible for the dark cocoon color, which protects immature wasps in the cocoons from detrimental sunlight^50^. Finally, the difference in hardness between the two areas of the cocoon may be attributed to differences in the thickness rather than those in the density and chemical composition; the ridge was thicker and slightly higher in sulfur and calcium. Although calcium oxalate contributed to cocoon strength in a moth^51^, we did not find strong evidence to support the presence of calcium as calcium oxalate. The central ridge not only adds physical strength to the whole body of the cocoon, but may also function to increase rolling distance after landing. This hopping behavior is metabolically expensive and increases the amount of carbon dioxide, humidity, and heat within the cocoon, necessitating proper ventilation through the cocoon shell for respiration, thermoregulation, and water vapor balance^52–56^. While the brown body may serve as a protective barrier against radiation of shortwave lengths^57^, the central ridge may allow ventilation and light transmission through the gap between fibers.

This study specified the wavelengths avoided by the jumping cocooned larvae of *B. anurus* and revealed the microstructural, chemical, and physical differences between central ridge and main body of the cocoon shell. Although the intensity of streetlights (7–15 lux^28,58^) is lower than the level tested in the current study (approximately 780 lx), heavy traffic at night could bring about light pollution and requires caution when light-sensitive biological control agents with limited dispersal ability as *B. anurus* larvae and pupae are released to be spread in suburban and urban settings.

## Supplementary Information

Supplementary Figures.

## Data Availability

The datasets generated during and/or analyzed during the current study are available upon reasonable request (M. Tuda: tuda@grt.kyushu-u.ac.jp).
