# Correction: Negative phototaxis of jumping cocooned parasitoid wasp larvae against short wavelengths and physicochemical properties of the cocoon shell

**DOI:** 10.1038/s41598-025-22552-8

**Published:** 2025-10-14

**Authors:** Shun‑ichiro Iwase, Midori Tuda, Yuma Sugawara, Katsuto Fukuda, James R. Miksanek, Midori Watanabe

**Affiliations:** 1 Institute of Biological Control, Faculty of Agriculture, Kyushu University, Fukuoka, 819‑0395 Japan; 2 Laboratory of Insect Natural Enemies, Department of Bioresource Sciences, Faculty of Agriculture, Kyushu University, Fukuoka, 819‑0395 Japan; 3 Center of Advanced Instrumental Analysis, Kyushu University, Fukuoka, Japan; 4 Present Address: Research Institute of Environment, Agriculture and Fisheries, Habikino, Osaka Prefecture Japan

Correction to: *Scientific Reports* 10.1038/s41598-023-36686-0, published online 12 June 2023.

The original version of this Article contained an error in the Abstract.

“The distance from the boundary to the cocoons in the shaded area was longer under these long wavelengths, followed by the red light and shortest under the near-infrared light and nil under darkness.”

now reads:

“The distance from the boundary to the cocoons in the shaded area was longer under these short wavelengths, followed by the red light and shortest under the near-infrared light and nil under darkness.”

The original Article has been corrected.

