# Supplementary material for: Negative phototaxis of jumping cocooned parasitoid wasp larvae against short wavelengths and physicochemical properties of the cocoon shell
Source: Sci Rep. 2023 Jun 12;13:9562. doi: 10.1038/s41598-023-36686-0 (PMC10261109; doi:10.1038/s41598-023-36686-0)
Supplement: Supplementary file 1 — Supplementary Figures. [file 41598_2023_36686_MOESM1_ESM.docx]

Supplementary materials

Negative phototaxis of jumping cocooned parasitoid wasp larvae against short wavelengths and physicochemical properties of the cocoon shell

Shun-ichiro Iwase^1$^, Midori Tuda^1,2*^, Yuma Sugawara^1^, Katsuto Fukuda^1^, James R. Miksanek^1^, Midori Watanabe^3^

^1^Institute of Biological Control, Faculty of Agriculture, Kyushu University, Fukuoka, Japan

^2^Laboratory of Insect Natural Enemies, Department of Bioresource Sciences, Faculty of Agriculture, Kyushu University, Fukuoka, Japan

^3^Center of Advanced Instrumental Analysis, Kyushu University, Fukuoka, Japan

*tuda@grt.kyushu-u.ac.jp

$Present affiliation, Research Institute of Environment, Agriculture and Fisheries, Osaka Prefecture,

Japan

Figure S1. Spatial distribution of sulfur and calcium on the surface of a *Bathyplectes anurus* cocoon.

Figure S2. FTIR results of different regions (brown main body and white central ridge) of *Bathyplectes anurus* cocoon surface, compared to the unprocessed cocoon silk and calcium oxalate.
